# RNA-seq reveals the diverse effects of substrate stiffness on epidermal ovarian cancer cells

**DOI:** 10.18632/aging.103906

**Published:** 2020-10-22

**Authors:** Xiaoxu Yang, Guohui Wang, Xiaolei Huang, Min Cheng, Yangyang Han

**Affiliations:** 1 School of Life Science and Technology, Weifang Medical University, Weifang 261053, Shandong, P.R. China; 2 Department of Physiology, Weifang Medical University, Weifang 261053, Shandong, P.R. China

**Keywords:** epidermal ovarian cancer, metastasis, RNA-seq, substrate stiffness

## Abstract

Background: Increasing evidence has confirmed that ovarian cancer is a mechanically responsive tumor both *in vivo* and *in vitro*. However, an understanding of the complete molecular mechanism involved in the response to substrate stiffness is lacking, as the associated transcriptome-wide effects have not been mapped. This limited understanding has restricted the identification of potential mechanically responsive targets in ovarian cancer.

Results: To address these limitations, we used a polyacrylamide hydrogel system with a tunable Young’s modulus that broadly ranged from soft (1 kPa) to normal (6 kPa) and stiff (60 kPa) and investigated the effect of substrate rigidity on the morphology, spreading area, and cytoskeleton of SKOV-3 epidermal ovarian cancer (EOC) cells. RNA-seq analysis of these cells was then performed at appropriate timepoints to map the transcriptome-wide changes associated with stiffness sensing. We identified a large number of stiffness-sensing genes as well as many genes that were enriched in cancer-related pathways. Informed by these diverse expression results and based on bioinformatics analysis, we evaluated the hypothesis that PLEC and TNS2, which are located in focal adhesions and regulated by lnc-ZNF136, may play key roles in the EOC response to substrate stiffness.

Conclusion: Overall, the results of the present study reveal previously unknown features of the EOC stiffness response and provide new insights into EOC metastasis in the clinic.

## INTRODUCTION

Ovarian cancer accounted for an estimated 295,414 new cases and 184,799 deaths in 2018 worldwide [1]. Ovarian cancer can be classified into three large groups, epithelial, germ cell, and specialized stromal cell tumors [2], with more than 90% of malignant ovarian tumors being epithelial in origin in clinical cases [3]. At present, there is a lack of efficient methods for screening and early diagnosis of this disease. Consequently, and due to an absence of early warning symptoms, approximately 70% of cases are diagnosed at an advanced stage and have a poor prognosis, with a 5-year relative survival rate of only 29% [4].

Migration to the abdominal cavity is frequently observed in ovarian cancer patients and is a major cause of unfavorable outcomes and poor prognosis [5]. However, it is extremely difficult to overcome and control this metastasis [6]. Despite ongoing basic research, the detailed molecular mechanism of metastasis in ovarian cancer remains unknown. Thus, there is an urgent need to elucidate the molecular mechanisms associated with this process, which may ultimately improve patient outcomes.

Recent evidence has demonstrated that cancer cells can interpret and respond to the mechanical properties (*e.g*., stiffness and topology) of the extracellular matrix (ECM) [7, 8]. The translation of mechanical forces into biochemical signals has been shown to regulate nearly every facet of cellular life, including shape, migration, survival, proliferation, and differentiation [9–11]. This phenomenon has been particularly well-studied in breast cancer [12, 13], liver cancer [14, 15], and pancreatic cancer [16, 17], wherein tumor progression is characterized by progressive stiffening. For example, Malik et al. demonstrated that substrate rigidity controls human desmoplastic matrix anisotropy to enable pancreatic cancer invasion through extracellular signal-regulated kinase 2 [18].

Several studies have indicated that ovarian cancer development involves mechanical stimuli, although the molecular mechanisms responsible for the translation of mechanical forces into biochemical signals remain largely unexplored to date. Some clinical results have confirmed that most malignant lesions of the ovary are soft and have a very small strain ratio compared with normal lesions [19, 20], and low-grade serous ovarian carcinomas are stiffer than high-grade lesions in *vivo* [21]. However, in cells, there are many similarities in the progression between breast and ovarian cancer, *e.g.*, the levels of both lysyl oxidase 1 (LOX1), a collagen-crosslinking enzyme that is crucial for matrix stiffness in breast cancer [22], and tissue transglutaminase (TG2), which also promotes ECM polymerization and crosslinking [23], are increased in ovarian cancer. These findings strongly indicate similarities in the molecular mechanical response mechanism between the two cancers.

In the present study, we used a polyacrylamide hydrogel system with a tunable stiffness range from soft (1 kPa) to normal (6 kPa) and stiff (60 kPa). Cell morphology, spreading area and cytoskeleton (F-actin) of epidermal ovarian cancer (EOC) cells (SKOV-3) were evaluated under the three substrate stiffness conditions, and an appropriate timepoint (24 h) with significant differences among the three substrate stiffnesses was chosen for RNA-seq analysis to elucidate the broad changes in the human EOC cell transcriptome as a function of stiffness. To investigate the possible mechanically responsive mechanisms of SKOV-3 cells, our sequencing data and that obtained from the GEO and TCGA databases were used for bioinformatics analysis. The results showed that SKOV-3 cells responded to different stiffness ranges in various ways, and we hypothesized that two genes, TNS2 and PLEC, both of which were regulated by lnc-ZNF136, may be the major responsive genes in the metastatic process of EOC cells in response to substrate rigidity. Overall, our results expand on the understanding of how cells mechanically respond to substrate stiffness by providing insights into the broad transcriptional changes in EOC cells induced by changes in substrate stiffness.

## RESULTS

### Cell area, morphology and cytoskeleton are correlated with substrate rigidity

**Figure 1:**
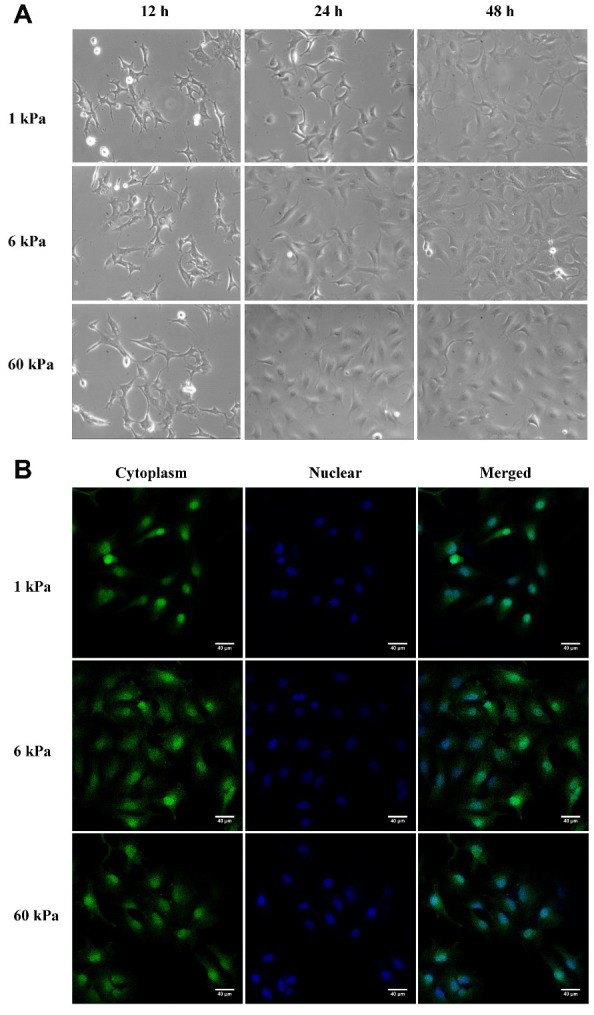
**Cell morphology closely correlate with substrate rigidity.** (**A**) Typical DIC images of SKOV-3 cultured on polyacrylamide hydrogels with different substrate rigidity. SKOV-3 cells were plated on collage I-coated 1 kPa, 6 kPa and 60 kPa hydrogels, and then images were taken at 12 h, 24 h or 48 h. (**B**) SKOV-3 cells plated on collage I-coated 1 kPa, 6 kPa and 60 kPa hydrogels for 24 h were fixed and stained with CellTracker fluorescent probes (left) and DAPI (middle) to visualize cytoplasm and nucleus respectively. Scale bar - 40 μm.

The transcriptional response to stiffness was profiled in SKOV-3 EOC cells during growth on polyacrylamide hydrogels. Cells were cultured in hydrogels at three stiffnesses spanning a range shown to influence cellular morphology (Figure 1). However, compared with the DIC images (Figure 1A), cells in Figure 1B which were stained using CellTracker fluorescent probes seemed to have the largest spreading areas of 6 kPa compared with 1 kPa or 60 kPa. Considering the introduction of CellTracker and DIC overview, one possible reason is as cell spreading area increased with the increasing of PA gels rigidity, the cells became thinner, resulting in less cytoplasm around the cells, which was not easy to be detected by dye staining.

**Figure 2:**
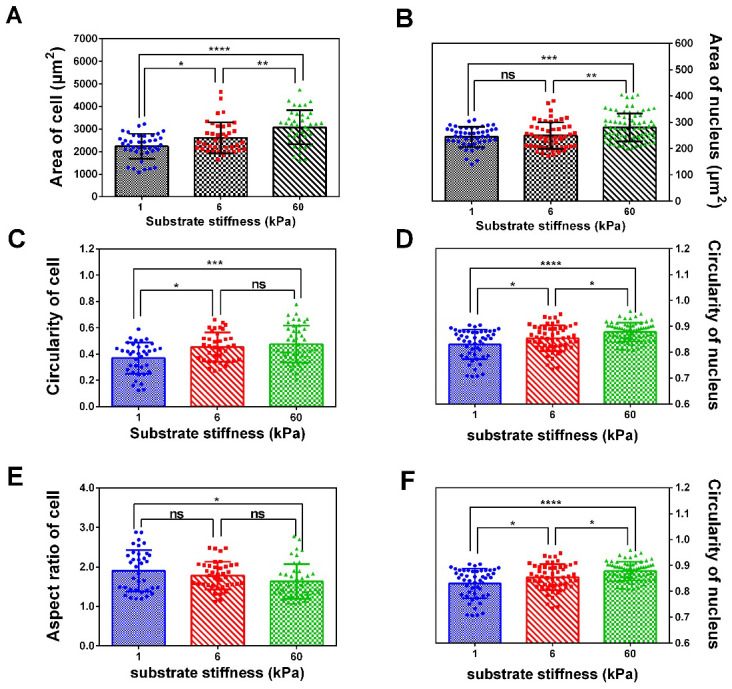
**Spread area, circularity and aspect ratio of cell and nucleus correlate with substrate rigidity.** Spread area (**A** and **B**), circularity (**C** and **D**) and aspect ratio (**E** and **F**) of SKOV-3 cells and nucleus were analyzed using ImageJ software. Each column represents means ± SE of 40-60 cells from 2 independent experiments. *P < 0.05, ***P <0.001, ****P < 0.0001.

After 24 h of cultivation, the spreading areas of SKOV-3 cells and nuclei showed a significant increase as the substrate stiffness increased from 1 kPa to 6 or 60 kPa (Figure 2A, 2B). Moreover, both the circularity (4πA/perimeter^2^) of the cells and nuclei exhibited significant improvements as the stiffness increased from 1 kPa to 6 or 60 kPa (Figure 2C, 2D), as the aspect ratio (long-axis length/short-axis length) of cells and nuclei simultaneously decreased (Figure 2E, 2F), confirming that not only the spreading area but also the morphology of the cells was closely correlated with the substrate rigidity.

**Figure 3:**
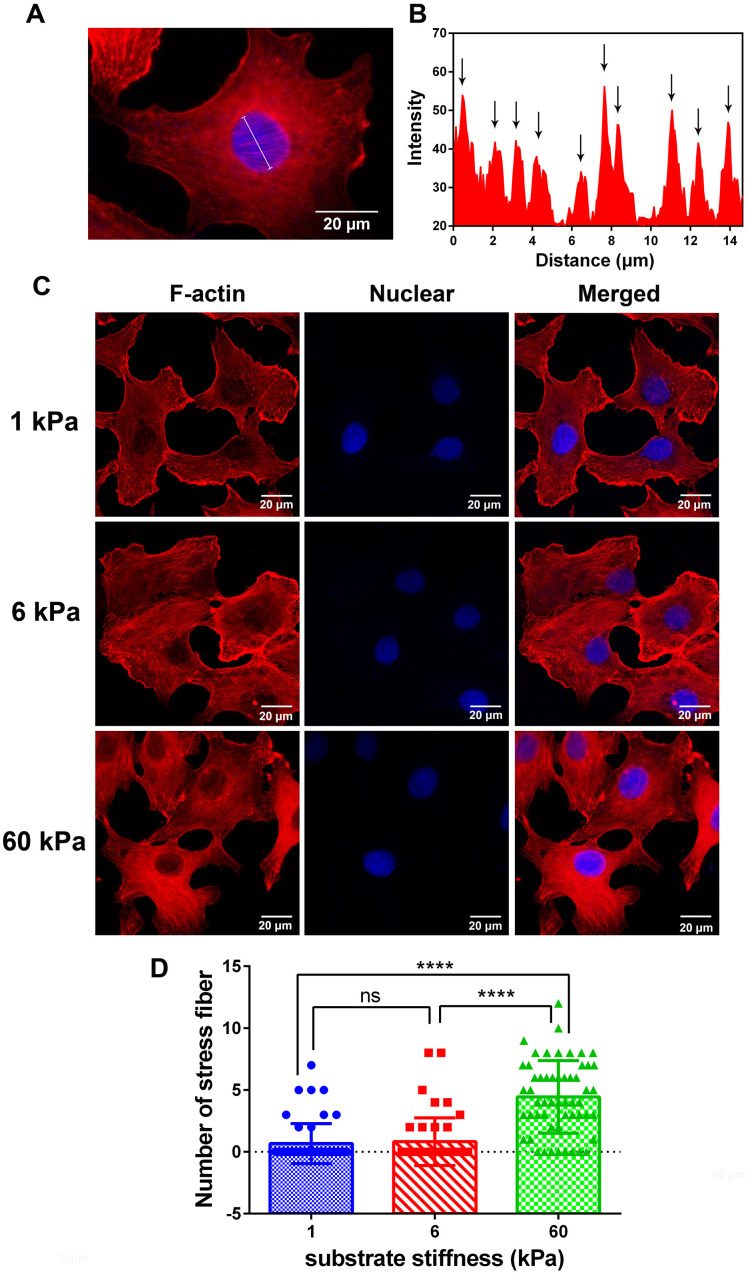
**Immunofluorescence images and analysis of F-actin in SKOV-3 cells which grown on substrate with different rigidity.** (**A**) Typical image was presented for defining the perinuclear actin stress fiber, and its analytic method using intensity calculated by ImageJ software (**B**). (**C**) SKOV-3 cells plated on collage I -coated 1 kPa, 6 kPa and 60 kPa hydrogels for 24 h were fixed and stained with rhodamine phalloidin (left) and DAPI (middle) to visualize F-actin and nucleus respectively. Scale bar - 20 μm. (**D**) Numbers of perinuclear actin stress fiber in SKOV-3 cells which grown on collage I-coated 1 kPa, 6 kPa and 60 kPa hydrogels for 24 h. Each column represents means ± SE of 49-55 cells from 2 experiments. **** *P* < 0.0001.

Actin stress fibers are bundles composed of actin filament, and play an important role in connecting the intracellular microenvironment to extracellular microenvironment, Here, we also quantified the effect of substrate rigidity on stress fibers, observing that perinuclear actin stress fibers were significantly influenced by culturing SKOV-3 on hydrogels for 24 h (Figure 3). As shown in Figure 3A, actin stress fibers were detected based the intensity which calculated by ImageJ software (Figure 3B), and typical images of SKOV-3 cells grown on different substrate rigidities and stained with rhodamine phalloidin and DAPI are shown in Figure 3C. The number of stress fibers significantly increased as the substrate rigidity increased from 1 or 6 to 60 kPa, while there was no significant difference between the 1 and 6 kPa treatments (Figure 3D).

### RNA-seq of SKOV-3 cells and quality control

**Figure 4:**
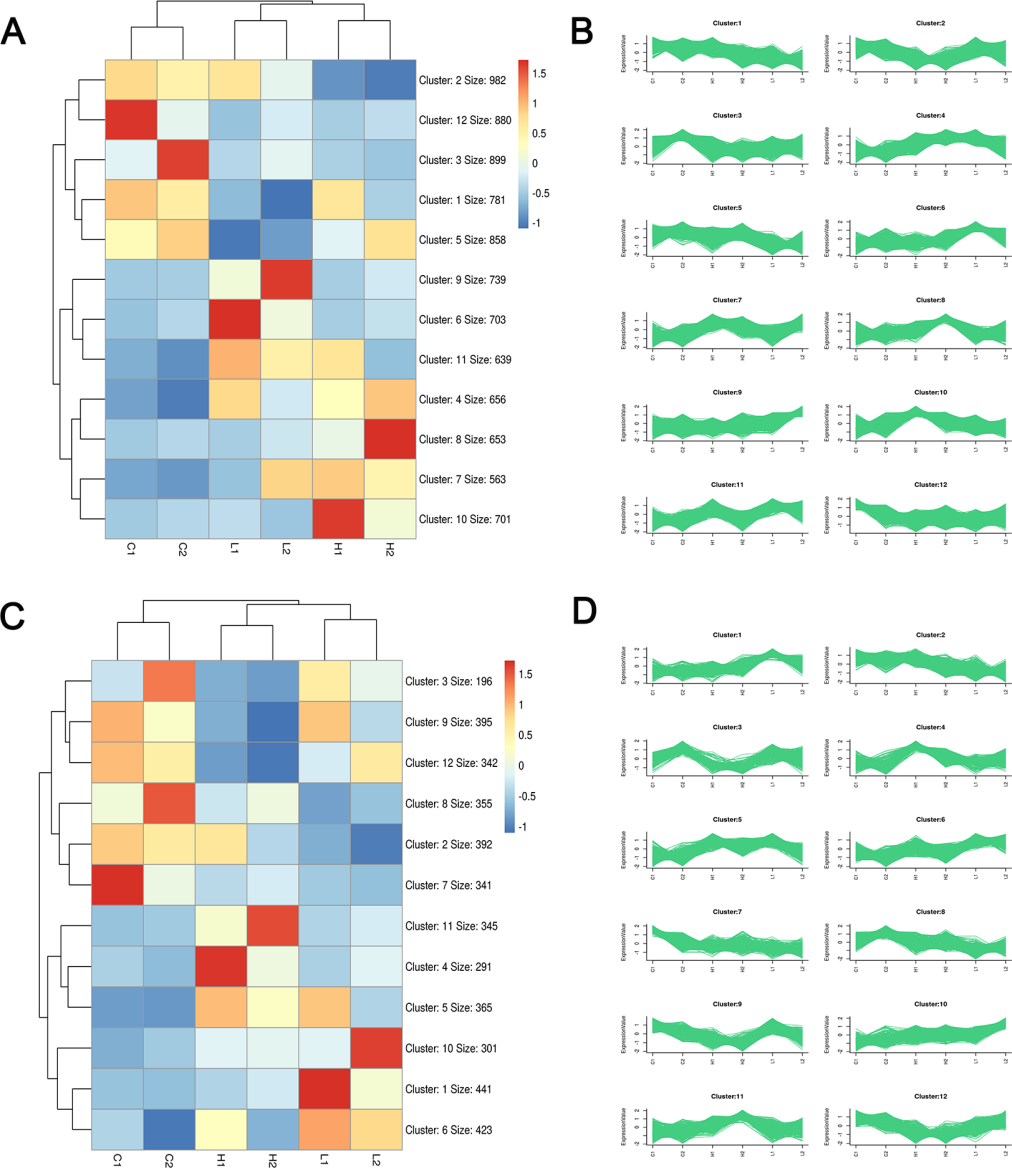
**Clustering analysis plots based on Kmeans pattern recognition algorithm.** Heatmap and sequence diagram of mRNA (**A**, **B**) and LncRNA (**C**, **D**) were obtained through EOC sample sequencing data analysis, respectively. Red, blue, and white colors respectively represent the relatively high, low, and equal expression.

Considering the results shown in Figuires 1–3, we cultured cells for 24 h and performed RNA-seq to identify the early events involved in stiffness sensing by SKOV-3 cells in a global and unbiased manner to generate hypotheses regarding the effects of stiffness sensing. After quality control, the RNA-seq data were determined to be of high quality and saturation (Suplementary Table 1 and Supplementary Figure 1). Clustering analysis based on the kmean pattern recognition algorithm showed the same group and different group expression patterns in 12 clusters, respectively, suggesting the unity within the group and comparability among the control, high and low groups (Figure 4).

### Differential gene expression data collection

**Figure 5:**
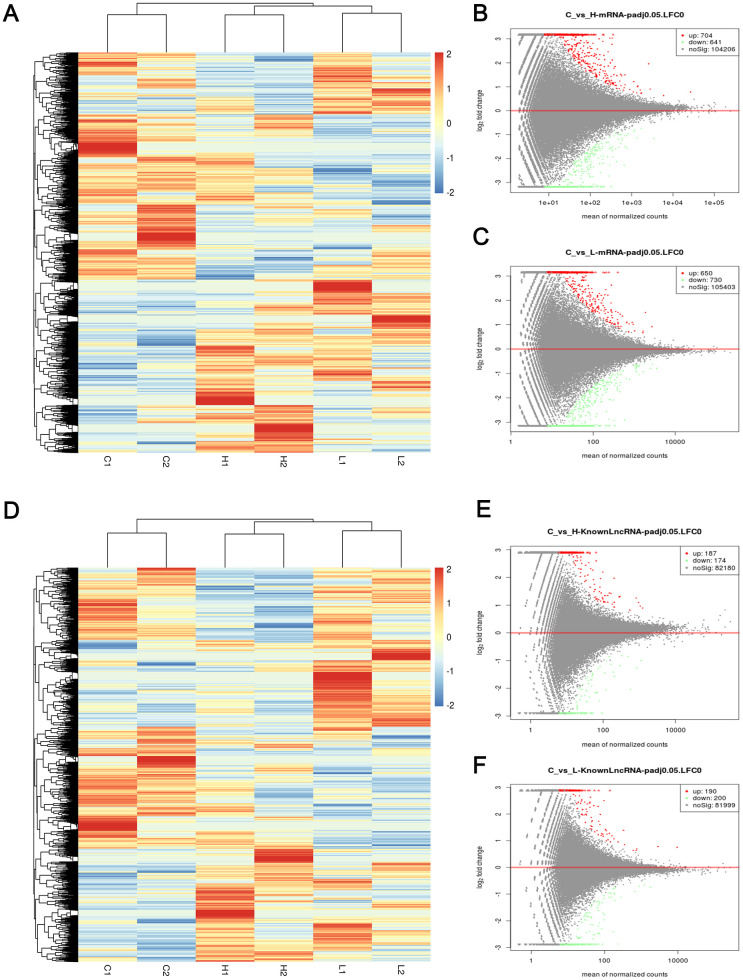
**Heatmap and volcano maps of DEMs and DELs.** (**A**) Heatmap based on mRNA sequencing data of 6 samples; (**B**, **C**) volcano maps based on mRNA sequencing data of group C versus group H (up-regulated:704, down-regulated: 641) and group C versus group L (up-regulated:650, down-regulated: 730); (**D**) Heatmap based on LncRNA sequencing data of 6 samples; (**E**, **F**) volcano maps based on LncRNA sequencing data of group C versus group H (up-regulated: 187, down-regulated: 174) and group C versus group L (up-regulated: 190, down-regulated: 200). Red, blue, and white colors respectively represent the relatively high, low, and equal expression in heatmap, while green, red, and black colors respectively represent relatively low, high, and equal expression in volcano maps. Group C, L and H represent control (6 kPa), low (1 kPa) and high (60 kPa) group, respectively.

The sequencing results for the whole transcriptome revealed that set a and set B include 1101 and 1082 DEMs, respectively. Additionally, 390 differentially expressed lncRNAs (DELs) were screened out between the control and low groups and 361 between the control and high groups (p value <0.05). A heatmap and volcano maps of DEMs and DELs showed that these lncRNAs and mRNAs were significantly differentially expressed between the case and control samples (Figure 5). The EOC-associated mRNA expression data and clinical data for 451 samples were obtained from the TCGA database. After screening based on the cut-off criteria, we obtained 4463 DEMs (Supplementary Figure 2). In addition, we used GEO2R to compare samples with versus without metastases in GEO (GSE30587) and identified 1260 DEMs in this dataset.

### Network construction and pathway enrichment of markers of EOC substrate stiffness

**Figure 6:**
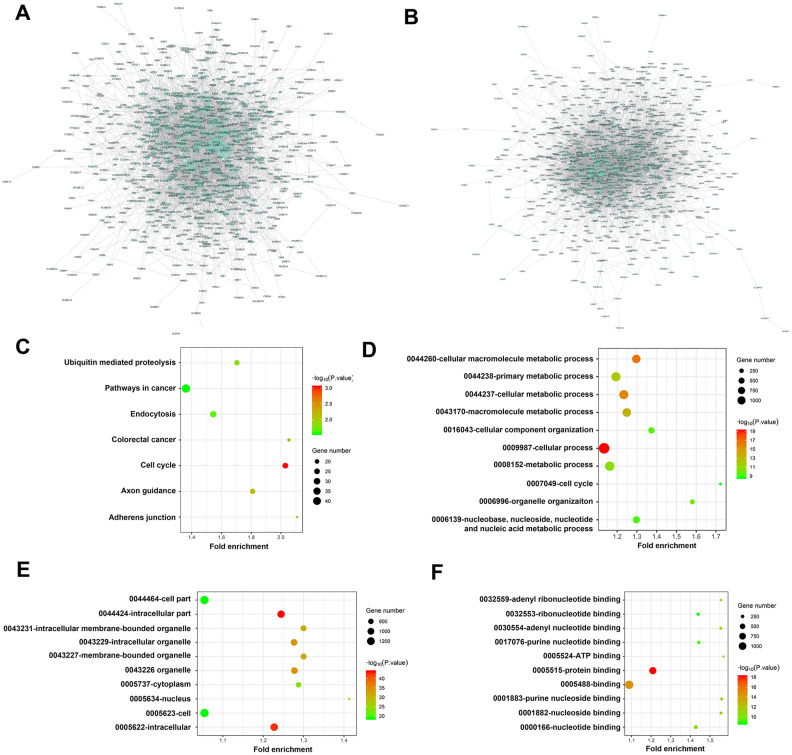
**Visualization analysis of PPI network, KEGG pathways and GO terms.** (**A**) Network of 1101 DEMs between C and L group (917 nodes and 4735 edges); (**B**) Network of 1082 DEMs between C and H group (800 nodes and 3738 edges); KEGG pathway enrichment was shown in (C) biological process, cellular component and molecular function terms were shown in **D**, **E**, **F**, respectively. The cutoff criterion was p < 0.05, and the first 10 pathways are shown. Group C, L and H represent control (6 kPa), low (1 kPa) and high (60 kPa) group, respectively.

PPI network was performed after excluding redundant unassociated nodes, a network of 1101 DEMs between the control and low groups (917 nodes and 4735 edges) and 1082 DEMs between the control and high groups (800 nodes and 3738 edges) was constructed and analyzed (Figure 6A, 2B).

Besides, the results of the KEGG pathway enrichment analysis showed that these DEMs were significantly enriched in cancer-associated pathways, demonstrating a high correlation with tumor progression (Figure 6C). The GO results showed that these DEMs were primarily involved in cell cycle and organelle organization at the biological process (BP) (Figure 6D) and cellular component (CC) levels, with the nucleus being the most significant category (Figure 6E). Additionally, at the molecular function (MF) level, adenyl ribonucleotide binding, adenyl nucleotide binding, ATP binding, purine nucleotide binding and nucleotide binding were significantly enriched (Figure 6F).

### Identification and characteristic analysis of PLEC and TNS2 enriched in the focal adhesion signaling pathway

**Figure 7:**
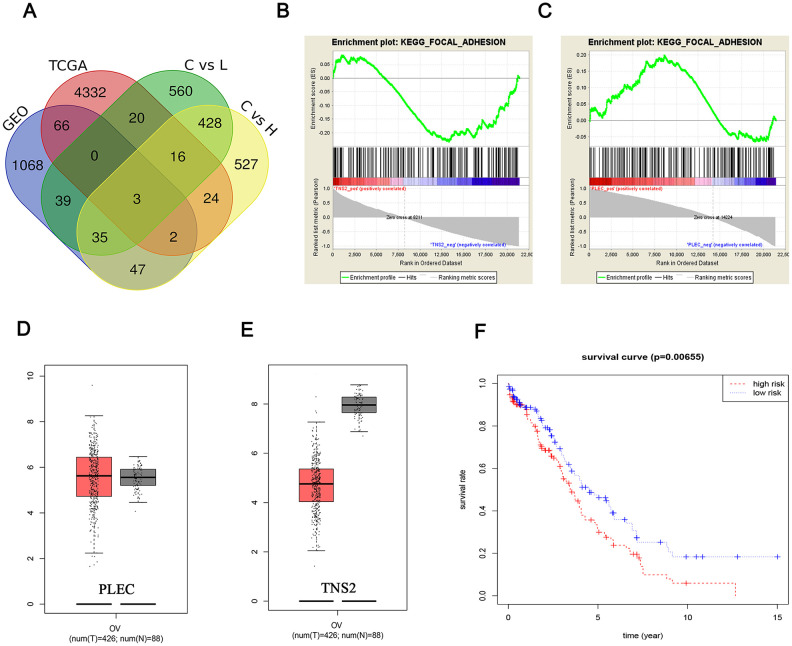
**Fishing and characterization of key targets.** (**A**) Venn diagram of the DEMs from four data arrays. A total of 3 significantly overlapping mRNAs were finally obtained; Identification of the enriched gene sets with GSEA analysis focused on a single gene as a phenotype in the merged microarray. Over-representation of negative TNS2 (**B**) and positive PLEC (**C**) were associated with focal adhesion pathway; PLEC (**D**) expression was upregulated in breast tumors compared with that in normal mammary tissues, while the TNS2 (**E**) expression was on the contrary. Red and black colors respectively represent tumor and normal samples. The total sample was 514, of which the number of tumor samples and normal samples were 426 and 88, respectively; (**F**) The differences of TNS2 and PLEC cor-survival trend between the high-risk and low-risk groups were determined by the log-rank test.

TNS2, PLEC and MGRN1 were identified through a Venn analysis (Figure 7A). To evaluate key cellular processes and signaling pathways associated with the changes in substrate stiffness, differential gene regulation was analyzed by GSEA. Interestingly, we observed that the differential regulation of PLEC and TNS2 was significantly enriched in the focal adhesion signaling pathway (Figure 7B, 7C), and the ES values of different trends also showed different expression patterns of the two genes. Focal adhesion is considered crucial in EOC because it not only regulates the internal environment but is also closely associated with EOC metastasis. Consequently, we focused further on focal adhesion in subsequent research examining the key targets TNS2 and PLEC.

Based on analysis of sample data from the TCGA and GTEx databases (426 tumor and 88 normal) in GEPIA (http://gepia.cancer-pku.cn), the upregulated of PLEC and downregulation of TNS2 in tumor tissues was found, which further validated the expression trend of the GSEA results (Figure 7D, 7E). In addition, the risk trends of PLEC and TNS2 were summarized, and the clinical data were further integrated to explore the survival analysis. The results showed that the overall low risk resulted in a longer survival time of EOC patients (p value <0.05) (Figure 7F).

### Determination of the regulatory relationship of lncRNA-mRNA targets based on coexpression and cis-trans analyses

**Figure 8:**
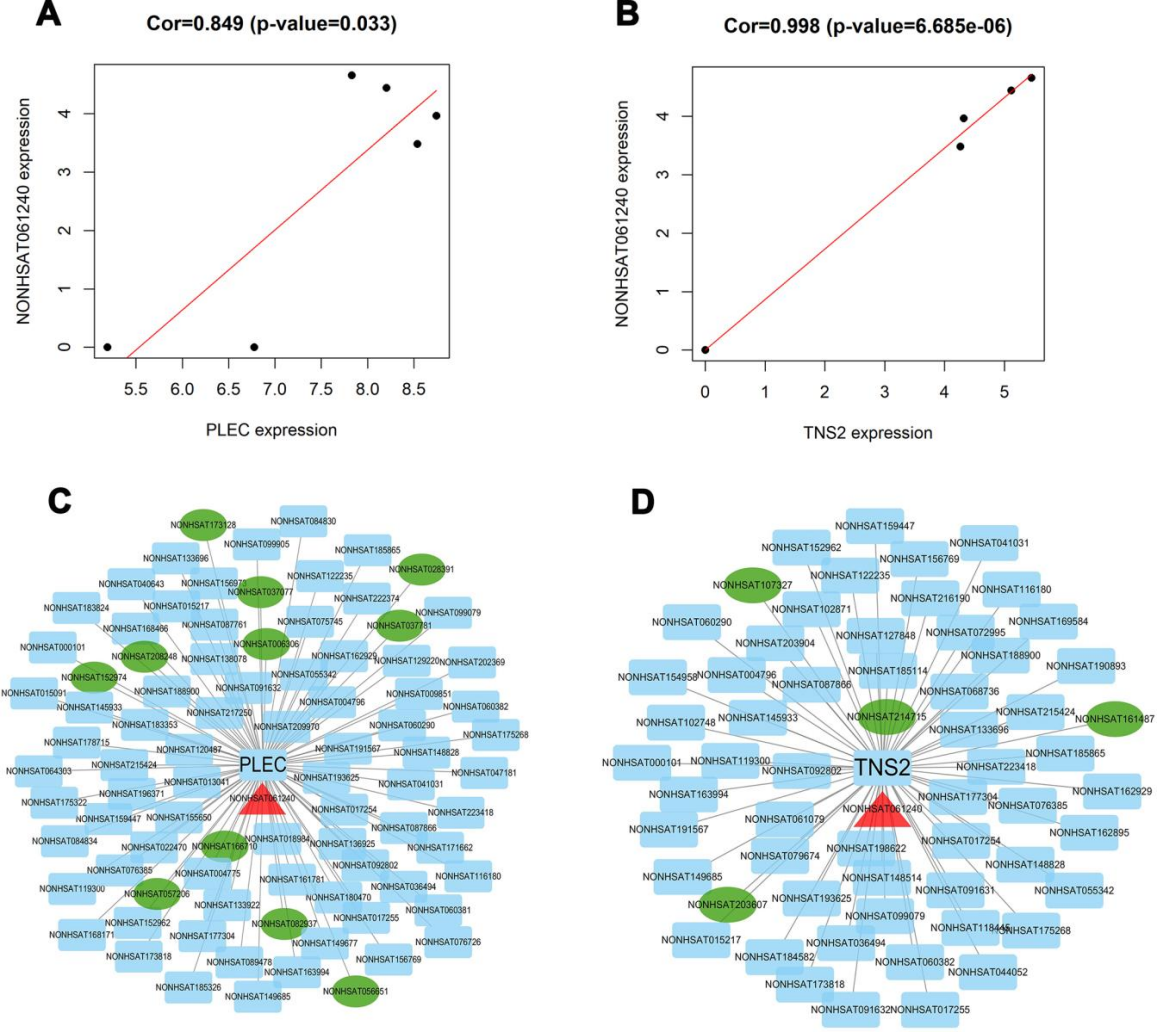
**Identification of key lncRNAs associated with TNS2 and PLEC.** (**A**, **B**) co-expression analysis was performed by using the Pearson correlation in R package. lncRNA NONHSAT061240 was found to be significantly associated with PLEC (score: 0.849) and TNS2 (score: 0.998); (**C**, **D**) Visualization network of two lncRNA-mRNA interaction pairs prediction mode. Except for TNS2 and PLEC at the center, the blue rectangle represents the associated LncRNAs obtained by the co-expression model, the green ellipse represents the associated LncRNAs obtained by the trans-cis model, and the red triangle represents the shared LncRNA.

Based on the related lncRNA and mRNA expression data, coexpression analysis was performed using the Pearson correlation. The results showed that 57 and 77 lncRNAs were highly correlated with TNS2 and PLEC, respectively (Supplementary Table 2, Figure 8A, 8B). Additionally, in another prediction mode of lncRNA-mRNA interaction pairs (cis and trans), 5 and 12 lncRNAs showed possible regulatory relationships with TNS2 and PLEC, respectively (Figure 8C, 8D). Interestingly, lncRNA NONHSAT061240 (lnc-ZNF136) was highly enriched in two regulatory modes, the details for which were obtained using the UCSC and LNCipedia databases, including the sequence information, length, exon number, and position, among others (Supplementary Table 3). Due to the novelty of this lncRNA, its disease correlation merits further characterization.

## DISCUSSION

The results of the present study demonstrated that SKOV-3 cells, a type of EOC cell, represent a mechano-responsive malignancy, and the transcriptome-wide response of these cells to substrate rigidity is significant and varied. The polyacrylamide hydrogels used in the present study have been widely shown to provide different substrate rigidities, thereby allowing for the study of the effect of substrate mechanical properties on some mechanisms associated with cancer, such as breast cancer [12, 26] and liver cancer [27]. However, related research on ovarian cancer has remained limited. A primary reason for this deficiency is that, in contrast to other mechano-responsive tumors, the positioning of the ovary deep within the abdominal cavity precluded the use touch to palpate and detect abnormalities until the emergence of magnetic resonance elastography (MRE) [28]. Using MRE to detect stiffness in seven clinical patients in *vivo*, the shear stiffness of the normal ovary is approximately 2 kPa [29]. Considering that Young’s modulus (*E*) and the shear modulus (*G*) are related by the Poisson ratio for isotropic homogeneous materials [*E*=2G*(1+*ν*)], typically using *ν ≈* 0.5 for tissues [30], the normal Young’s modulus of hydrogels is set as 6 kPa, while 1 kPa is softer and 60 kPa is stiffer than normal [24].

Using polymer hydrogels to create various substrate rigidities, we showed that substrate mechanical properties modulate the morphology (Figure 1A, 1B) and spreading area of SKOV-3 cells, including the whole cell (Figure 2A) and the nucleus (Figure 2B), as well as the circularity and aspect ratio (Figure 2C–2F). Consistent with the findings of Mckenzie et al., who used hydrogels with a Young’s modulus of 3 and 25 kPa and glass coverslips to culture EOC, they also confirmed that the mechanical microenvironment could regulate the morphology, migration, and spheroid disaggregation of ovarian cancer cells [25]. It is important to note that the elastic modulus values used herein to model and manipulate the mechanical microenvironment were based on detection in a clinical patient using MRE in *vivo* [29], while the settings used by Mckenzie et al. were based on analyses of murine peritoneum. Additionally, SKOV-3 cells grown on hydrogels with a 1, 6 or 60 kPa Young’s modulus not only showed an altered spreading area and morphology but also a significantly increased number of perinuclear F-actin stress fiber on 60 kPa compared with 1 or 6 kPa (Figure 3). F-Actin is a well-known component of the cell cytoskeleton, and some researchers have confirmed that it is involved in the response of cells to substrate rigidity in both 2D and 3D environments [31–33]. While actin stress fibers which composed of bundles F-actin are usually categorized into dorsal stress fibers, transverse arcs, and ventral stress fibers subtypes [34]. And it is well accepted that actin stress fibers form a continuous structural network that is mechanically coupled to the extracellular matrix through focal adhesions, thus play a major role in cell adhesion [35, 36], survival and proliferation [37]. Moreover, stress fibers at the periphery of the nucleus also plays a critical role in maintaining the mechanical stability of the nucleus [38]. Therefore, the increased actin stress fibers caused by the increased substrate stiffness may lead to a series of biological behavior changes in ovarian cancer cells, and it is worthy of further research.

Bioinformatics has been widely used in research and provides an unprecedented opportunity to expand our understanding of tumor-associated mechanisms [39, 40]. In the present study, RNA-seq analysis of the effects of substrate stiffness on SKOV-3 cultured in hydrogels generated a large number of integrated DEMs in our sequencing data. Based on the success of the hydrogel model, we performed RNA-seq, and after quality control, high-quality and saturated RNA-seq data were successfully obtained (Supplementary Figure 1). Then, based on the bioinformatics analysis results, the integrated DEMs were identified, and their enrichment pathways primarily correlated with ubiquitin-mediated proteolysis, cancer, endocytosis, colorectal cancer, cell cycle, axon guidance, and adherens junction pathways (Figure 6C). After further pathway enrichment analysis, two genes, PLEC and TNS2, became of interest due to their significant enrichment in the focal adhesion signaling pathway (Figure 7B, 7C). Focal adhesions are unique structures that form at the point of cell-extracellular matrix contact, where bundles of actin filaments are anchored to transmembrane receptors of the integrin family through multimolecular complexes connecting plaque proteins. The results of many studies have confirmed that focal adhesion is closely associated with metastasis in different types of cancers, such as cervical cancer [41], colorectal cancer [42], lung cancer [43], and ovarian cancer [44]. Thus, our results suggested that TNS2 and PLEC may play major roles in the metastasis of EOC.

TNS2 is a member of the Tensin family, members of which bind to actin filaments and participates in signaling pathways, and human TNS2 cDNA encodes a 1,285-aa sequence that includes actin-binding, Src homology 2 (SH2), and phosphotyrosine binding (PTB) domains [45]. Accumulating evidence has demonstrated that TNS2 plays a role in regulating cell proliferation and migration [45, 46], and it is downregulated in various cancers, including EOC (Figure 7E), which enhances tumorigenicity [47, 48]. PLEC encodes plectin, which is a versatile cytoskeletal linker protein of substantial size (>500 kDa) that is abundantly expressed in a wide variety of mammalian tissues and cell types. Homozygous or compound heterozygous mutations, the majority of which are loss-of-function mutations, have been identified in patients with epidermolysis bullosa simplex with muscular dystrophy [49, 50]. Additionally, some studies have indicated that PLEC may be a novel susceptibility gene in testicular cancer [51] and esophageal squamous cell carcinoma [52]. Taken together, these findings provide new insights suggesting that TNS2 or PLEC are novel mechanically responsive genes involved in cell metastasis associated with substrate stiffness in EOC and may be regulated by lncRNA NONHSAT061240 (lnc-ZNF136). Our future studies will examine these possibilities.

## MATERIALS AND METHODS

### Cell culture

The human epithelial ovarian cancer cell line SKOV-3 (ATCC) was maintained in a humidified incubator at 37°C under an atmosphere containing 5% CO_2_ in McCoy’s 5A medium (Thermo Fisher Scientific) supplemented with 10% fetal bovine serum (FBS).

### Preparation of polyacrylamide hydrogels

### Treatment of bottom coverslips or glass bottom dishes

After sterilization with 70% ethanol, coverslips (48 or 22 mm) or glass bottom dishes (Cellvis) were incubated with 0.1 M NaOH at room temperature for 1 h and then washed 4×10 min with ddH_2_O. Subsequently, the coverslips were incubated with 1% (v/v) 3-aminopropyltriethoxysilane (APTES; Johnson Matthey) at room temperature for 1 h, washed 2×10 min with ddH_2_O, and then incubated with 1% glutaraldehyde for 1 h. Activated coverslips were either used immediately or stored under desiccation for up to 2 weeks. The treatment protocol for glass bottom dishes was similar to that used to treat coverslips except that APTES and 0.5% glutaraldehyde treatments were performed for 10-15 and 30 min, respectively.

### Treatment of top coverslips

After being washed 3×5 min with ddH_2_O, the top coverslips were incubated with dimethyldichlorosilane (Sigma-Aldrich) for 5 min and then dried with an appropriate paper.

### Preparation of polyacrylamide gels

The polyacrylamide gels contained final concentrations of 3, 7.5 or 10% acrylamide and 0.1, 0.07 or 0.5% bis-acrylamide, which were chosen based on previously published values [24] to provide the desired Young’s elastic modulus values of 1, 6, or 60 kPa. Polymerization was initiated with 5 μl of 10% APS and 0.5 μl of N,N,N’,N’-tetramethyl-ethylenediamine (TEMED) in a final volume of 1 ml. Gels were cast onto activated bottom coverslips in glass bottom dishes by adding 400 μl of gel solution (for 48-mm coverslips) onto the activated surface, which was then overlaid with a smaller top coverslip (45 mm) that was also treated. Gels were allowed to polymerize for 15-20 min and then were soaked in ddH_2_O for more than 4 h after removing the top coverslips. Subsequently, the gels were incubated with 0.2 mg/ml sulfosuccinimidyl 6-(4’azido-2’-nitrophenylamino) hexanoate (sulfo-SANPAH; Thermo Fisher Scientific) for 25 min at room temperature under a 365 nm UV light source. The activated gels were washed 3×2 min in 50 mM HEPES (pH 8.5) and then incubated with 200 μg/ml of collagen I (Sigma-Aldrich) at 4°C for 7 h, after which they were washed 3×2 min with PBS and either immediately used or stored at 4°C up to 1 week. This protocol was selected based on its successful use to support SKOV-3 cell growth on gels, as it has been previously reported that these cells do not adhere to uncoated gels or to gels coated with bovine serum albumin [25].

### Immunofluorescence and confocal microscopy

SKOV-3 cells that had been grown on gels for 24 h were fixed in 4% formaldehyde in PBS for 10-15 min, washed 3×2 min with ddH_2_O, blocked with PBS containing 1% BSA for 1 h at room temperature and then incubated with rhodamine phalloidin (1:40, Invitrogen) containing 1% BSA for 40 min at 37°C. After being washed with PBS, the cells were incubated with Hoechst 33342 (1:500, Invitrogen) containing 1% BSA for 20 min at room temperature, and images were then captured using a confocal laser scanning microscope (LSM-710, Carl Zeiss AG, Germany).

### Cell morphological and stress fibers analysis

For the cell morphological analysis, cell counts were determined by staining the cytoplasm using CellTracker fluorescent probes (Thermo Fisher Scientific). Cell morphology was then evaluated using ImageJ (National Institutes of Health, Bethesda, MD, USA), including the area (A), circularity (4πA/perimeter^2^), and aspect ratio (long-axis length/short-axis length, under Fit Ellipse mode). Besides, number of actin stress fibers was analyzed based on the intensity calculated by ImageJ software.

### Cell retrieval from hydrogels

After 24 h of cultivation, SKOV-3 cells were washed with PBS 2×30 sec on ice and then digested with 0.25% trypsin-EDTA for 5 min at 37°C to ensure the removal of cells from the hydrogels. The cells were then centrifuged and rinsed twice before performing additional analyses.

### Data sequencing and extraction

Six groups of epithelial ovarian cancer cell samples for sequencing analysis, which were divided into the control group (C, 6 kPa, 2 samples), high group (H, 60 kPa, 2 samples) and low group (L, 1 kPa, 2 samples) with respect to the rigidity of hydrogels. For each sample, 3 μg RNA was used for RNA sample preparations. Ribosomal RNA was removed using Epicenter Ribo-Zero™ Gold kits (Human/Mouse/Rat/other) (Epicenter, USA). Subsequently, sequencing libraries were generated with different index labels using an NEBNext® Ultra™ Directional RNA Library Prep kit for Illumina (NEB, Ipswich, USA) following the manufacturer’s recommendations. For each library, the concentration of the input RNA was measured using a Qubit® RNA Assay kit with a Qubit® 2.0 fluorimeter for preliminary quantification before being diluted to 1 ng/μl. The insert size was assessed using an Agilent Bioanalyzer 2100 system (Agilent Technologies, CA, USA), and the qualified insert size was accurately quantified using a Taqman probe with the AB Step One Plus Real-Time PCR system (valid library concentration > 10 nM). The index-coded samples were clustered with a cBot cluster generation system using a TruSeq PE Cluster kit v4-cBot-HS (Illumina) according to the manufacturer’s instructions. After cluster generation, the libraries were sequenced on an Illumina platform, and 150-bp paired-end reads were generated.

Reads that included mRNAs and lncRNAs were mapped to the human genome using TopHat version 2.1.1 and HISAT2. Gene clustering analysis was performed using the kmeans pattern recognition algorithm based on R package ‘cluster’, and the comparability of each sample was judged by assessing the distribution of the gene clusters in the sample.

Besides, the gene expression and clinical data for EOC were downloaded from the TCGA database (https://cancergenome.nih.gov) and comprised 71 normal tissue samples and 380 tumor tissues. In addition, we conducted a systematic search of the GEO database (https://www.ncbi.nlm.nih.gov/gds) to identify EOC gene expression datasets with tumor metastasis. The gene expression data for GSE30587 (including 9 samples with and 9 samples without metastases) were obtained from the GEO database using “epithelial ovarian cancer” and “metastasis” as search keywords.

### Data processing and differential analysis

The goal of transcriptome research is to compare the changes in the expression (reads) of specific genes under different conditions. Differential expression analysis was performed using the R package ‘DESeq’, and differential expression analysis of genes regulated by substrate stiffness was conducted based on the negative binomial distribution pattern. Statistical tests based on negative binomial distribution patterns, such as those developed by Simon Anders and Wolfgang Huber, can be used to determine whether changes in the number of reads are greater than the expected number reads due to random variation. Raw count data were prepared using a custom Perl script based on the results obtained using eXpress software and imported into the DESeq framework. Information regarding the experimental design was also imported into the DESeq framework to form a count data set. Filtering was conducted to remove transcripts in the lowest 40% quantile of the overall sum of counts (irrespective of the biological condition) to increase the differential expression transcript detection rate. The Size Factor function estimate was used to estimate the effective library size to normalize the transcript counts. The Dispersions function estimate was used to estimate dispersion. The binom test function was used to ascertain the differential expression between two conditions. The false discovery rate (FDR) was controlled using the Benjamini-Hochberg method at an FDR of 5%. Besides, the EdgeR package, which is also based on the negative binomial distribution pattern, was used to analyze the differential expression of the TCGA data and to identify differentially expressed mRNAs (DEMs). Furthermore, to identify the key targets associated with epithelial ovarian cancer metastasis, the online analytical tool GEO2R was used to screen DEMs between samples with versus those without metastases in the GEO database.

All three sets of data met the cut-off criteria of P < 0.05. Due to slight difference in mRNA expression in terms of both substrate stiffness and metastasis condition samples, |log2fold change (FC)| ≥ 2 was only used to screen the TCGA data. Through the limitation of three transcription data sets (sequencing, TCGA and GEO data), we can obtain specific targets for EOC metastasis caused by different substrate stiffness.

### Protein-protein interaction (PPI) network construction and pathway enrichment analysis

Since proteins not only function individually but also cofunction with their interaction partners, an interaction network of genes was constructed and used for further causal studies. The Search Tool for the Retrieval of Interacting Genes (STRING) database is an online resource that provides protein-protein interaction (PPI) information by reporting both prediction and experimental observations. To analyze PPIs among the differentially expressed transcripts, we mapped the transcripts to nodes of PPIs in the STRING database (https://string-db.org/) via ID conversion. Interaction data were further evaluated using Cytoscape for visualization and bioinformatics analysis. Further, Gene Ontology (GO) and Kyoto Encyclopedia of Genes and Genomes (KEGG) enrichment analysis, which based on DAVID database (https://david.ncifcrf.gov), revealed the biological functions and pathways of the DEMs.

### Target searches for substrate stiffness and ovarian cancer metastasis

DEMs from the control vs low group and control vs high group comparisons were defined as set A and B, respectively, representing the key targets regulating substrate stiffness. Additionally, the DEMs screened from the TCGA were considered the key targets for regulating the occurrence and development of epithelial ovarian cancer and were defined as set C. DEMs screened from the GEO database were considered the key targets of epithelial ovarian cancer metastasis and were defined as set D. The differential mRNAs obtained by the intersection of the four sets were considered the direct regulatory markers regulating substrate stiffness and causing epithelial ovarian cancer metastasis.

### Gene set enrichment analysis (GSEA) and characterization of key targets

Gene set enrichment analysis (GSEA) is a computational methodology used to identify key pathways and gene sets between key targets and potential biological mechanisms. In the present study, we used GSEA version 4.0, which was downloaded from the official website. The Pearson method was used to evaluate the correlation between the expression of each gene and key targets, after which gene sets and pathways were ranked according to the correlation score. Gene set permutation was run 1000 times per analysis, and the normalized enrichment score (NES) and nominal P value were used to identify the pathways enriched in every phenotype. We further studied the survival analysis and expression state of the key targets based on sample data from the TCGA and GTEx databases.

### Coexpression method for the prediction of lncRNA-related mRNAs

To better evaluate the function of the relevant lncRNAs, we used the above key targets to predict and screen the lncRNA data. We identified lncRNAs that targeted key mRNAs and then analyzed the presence of any overlap among these lncRNAs. This step was performed based on coexpression analysis and trans-cis action mechanisms. LncRNAs can function within their neighboring genomic environment (in cis) and diffuse to distant sites of action (in trans), which is collectively known as the trans-cis action mechanism. Gene regulation may occur in cis or trans. We used the expression data and location information data for lncRNA and protein-coding genes to establish the lncRNA-mRNA action pair relationships. Additionally, the coexpression analysis used the Pearson correlation coefficient, which can directly reflect the linear correlation degree of the variables. The correlation test standard between lncRNA and differential mRNA expression was a p value <0.05.

### Statistical analysis

Orange statistical software was used to quantitatively analyze the data. The data are presented as the means ± SD. One-way ANOVA was performed to determine the statistical significance of differences among 2 or 3 orientations. Statistical significance was also determined using Student’s t-test (SPSS 12.0) at 0.05, 0.01, 0.001 or 0.0001 probability levels.

## Supplementary Material

Supplementary Table 3

Supplementary Table 2

Supplementary Table 1

Supplementary Figures
