# Correction for: RNA-seq reveals the diverse effects of substrate stiffness on epidermal ovarian cancer cells

**DOI:** 10.18632/aging.203438

**Published:** 2021-08-15

**Authors:** Xiaoxu Yang, Guohui Wang, Xiaolei Huang, Min Cheng, Yangyang Han

**Affiliations:** 1 School of Life Science and Technology, Weifang Medical University, Weifan, Shandong, 261053, P.R. China; 2 Department of Physiology, Weifang Medical University, Weifan, Shandong, 261053, P.R. China

**Keywords:** correction

Original article: Aging. 2020; 12:20493–20511.  . https://doi.org/10.18632/aging.103906

**This article has been corrected:** The authors replaced panel **2F** of **Figure 2**. During the revision of the figure in accordance with the reviewer's comments, panel **2F** was misplaced. This alteration does not affect the results or conclusions of this work.

New **Figure 2** is presented below.

Additionally, the authors corrected the **Figure 7** legend, where they mistakenly used the term “breast tumors” instead of “ovarian tumors”. The correct **Figure 7** legend is:

**Figure 7. Fishing and characterization of key targets.** (**A**) Venn diagram of the DEMs from four data arrays. A total of 3 significantly overlapping mRNAs were ultimately obtained. Identification of enriched gene sets with GSEA focused on a single gene as a phenotype in the merged microarray. Over-representation of negative TNS2 (**B**) and positive PLEC (**C**) was associated with the focal adhesion pathway; PLEC (**D**) expression was upregulated in ovarian tumors as compared to normal ovarian tissues, while TNS2 (**E**) expression was the opposite. Red and black colors respectively represent tumor and normal samples. The total number of samples was 514, among which there were426 tumor samples and 88 normal samples. (**F**) Differences in the TNS2 and PLEC cor-survival trend between the high-risk and low-risk groups were evaluated using the log-rank test.

**Figure 2:**
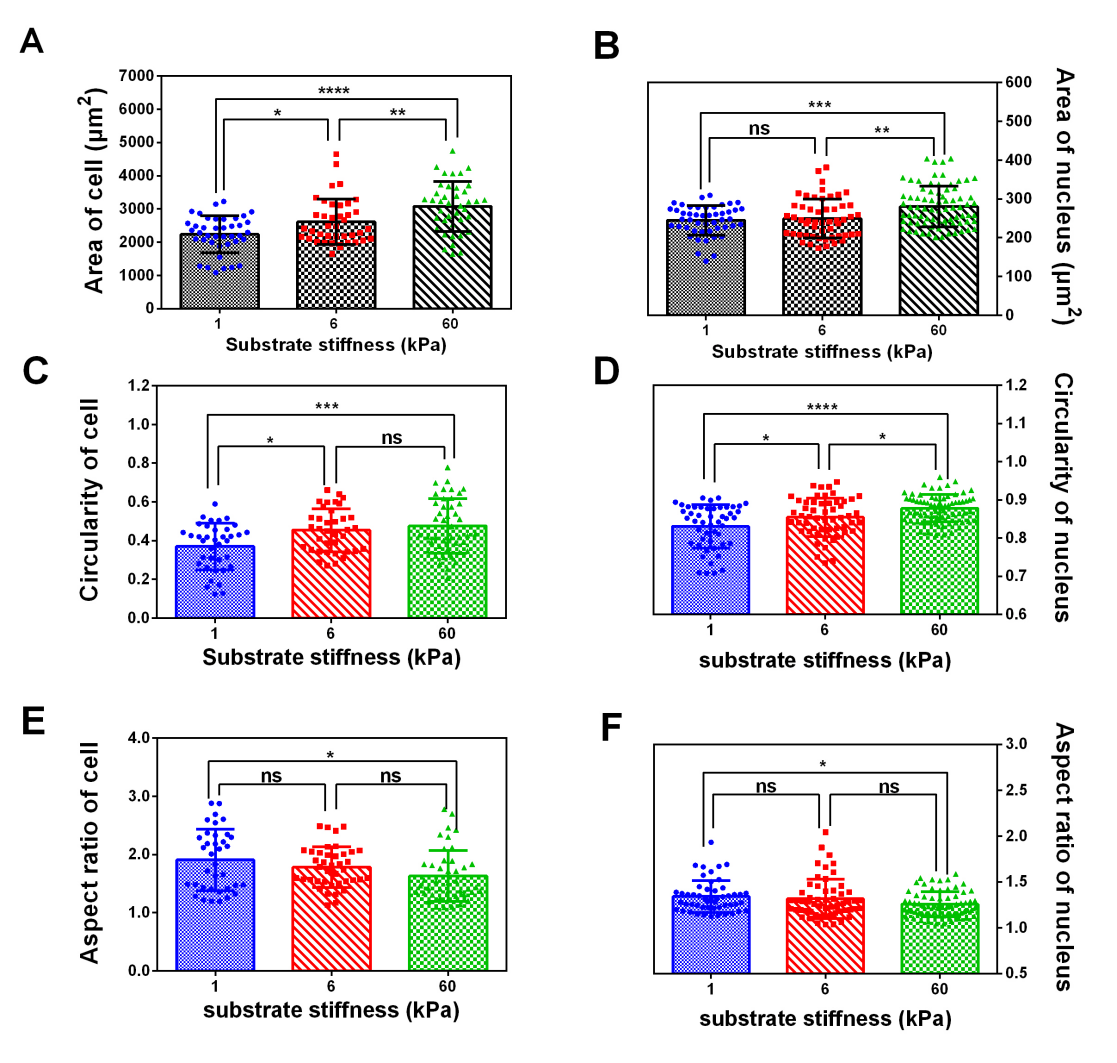
**The spread area, circularity and aspect ratio of cell and nucleus correlate with substrate rigidity.** Spread area (**A** and **B**), circularity (**C** and **D**) and aspect ratio (**E** and **F**) of SKOV-3 cells and nucleus were analyzed using ImageJ software. Each column represents the means ± SE of 40-60 cells from 2 independent experiments. *P < 0.05, ***P <0.001, ****P < 0.0001.

