# Supplementary material for: RNA-seq reveals the diverse effects of substrate stiffness on epidermal ovarian cancer cells
Source: Aging (Albany NY). 2020 Oct 22;12(20):20493–511. doi: 10.18632/aging.103906 (PMC7655203; doi:10.18632/aging.103906)
Supplement: Supplementary Table 3 [file aging-12-103906-s001..docx]

**Supplementary Table 3. Detailed informations of NONHSAT061240.**

NONHSAT061240

**Location (hg38):** [chr19:12234697-12239229](https://genome.ucsc.edu/cgi-bin/hgTracks?org=human&db=hg38&position=chr19:12234697-12239229&hubUrl=https://www.lncipedia.org/trackhub/hub.txt)

**Strand:** +

**Class:** antisense

**Transcript size:** 2268 bp

**Exons:** 2

**Alternative transcript names:** lnc-ZNF136-1:11

**RNA sequence:**

CATCACATCTCTACAGATTCTTCACGGAAAAATCCAGCAAAGCCTACTCCTTCTGGATGAAGTTCAGAGTCACATCTTCAAAGACCACCAAGCCGTGACATATCCCAGATATGTGTAGAGGAGGACAGGTGAAAGGCAGGATTGGGAACCCCACAGACCGCAGCTCCTCCCAGGAGAACCCCACACATGAGTCTGGATCCTCGCCATGATCCTCCCGTGGCCCCCACACAATCTGGGGAGACGCGGGGCCGCGGGCACTGCGCTGCCGAGAGGGCTCCGGACTGAGGCTGCAGTTGCTGCGCAGGGACGGCTCAGGATGCCCGGGGTCCCGGCTGCTGGCCCAGCCCCACCCTGTGGCCGAGGGGACCCAGGGACGAGCTGCGCCAGGGAGACTCGGGTCCGCAGACCCCGGAATCGCTGCTGACAGGCCCGGGTCCCACCACAGCAGGTCCCAACCAGCCCCTCCTCCCACCCGGCCTTGCACACTCACCATTTCCCGGCTCCCAGGTGTCCCGGGTCCTCCCTTGGCTCCGGAGATCAGTGCTGGTCACCACCGGACAGAAGCTGTGGTGGAGCCACCTCGGCCTCTGGAAGGCTGAATTCAGTGGGTTAGGGGAACAGGAGCCCCTTAGACTTGCGGAGCTTGGCCCCACCCTCCTGGCGGAGCGCCTGATTGGATAATTTACACGACCCCGCCCAGTACCCCTAGGATAGGAATCAAGCCCCACCCCCTAGGCCCGGGTTTCAGGAGAAGCCACGGAAAACTCGGAAGTGTGGAGCAGGATTGACAGGTTCTAGCAACAGCCCTCCCACCCCCCCGCCCAGGCCAGGCTTCCTTCTGGAGCTTGGACGTGACCCCACACTGGGGACATTTGCATTTAAGAAAAACTTGGCTCCAGGCTTTCCACAGTGGGCCCTGGCATCTTCCTATACTCACTGGGCCCTTTCTGTTTCTTCCTTCTGGACGGGGGTTCACAAGTGTGAGCAGGCAGTTCCACACTCCGTGTCCAATTGAGCCAGTACCTTTGCTGGAAGCTACTGGGCTGGAACAGGAGGGAGAGCCCAGATGTCTTCCAGGGATCAGGAATTTGGGATAAGCGGCTGGTGGCATGGCCAAGGCTTCCTCATACCTTATCTCAGTCTACCCATTTTCAGGGAAGAAAATGAAATATGCCAACAAGGGAACAAAAATTAAGTGCATGGAAAAAAGTTATACTACAGCAATATTCTGTAAAGAAATCAAAAGGGGCCGGGCGCGATGGCTCACCCTATAATCCCAGCACTTTGGGAGGCCAAGGTAGGAGGATCACCTGAGGTCAGGAGTTTGAGACCGCTCTGGCCAACATGGTGAAACCCCATTCTCTACTAAAAATACAAAAACTAGGCCAGGCGCGGTAGCTCACGCCTGTAATCCTAGCACTTTGGGAGGCCAAGGCGGGCGGACCACAAGGTCAAGAGATCAAGACTATCCTGGCCAACATGGTGAAACCCTGTCTCTACTAAAAATACCAAAAAAATCAGCTGGGCGTGGTGGCACGTGCCTGTAGTCAGAGCAGCTCGGGAGGTTGAGGCAGTAGAATTGCCTGAACCCGGGAGACAGAGGTTGCAGTGAGCCAAGATCACACCACTGCACTCCAGCCTGGCAACAGAGAGAGACTGTCTCAAAAAAAAAAAAAAAAAAAAAAATAGCTGGGCATGGTGGCACGTGCCTGTAATCCCAGCTACTCAGGAGGTCGAGGCAGGACAATCACTTGAACTCGGGAGGCGGAGGTTGCAGTGAGCCGAGATCGTGCCACTGCACTCCAGCCTGGGCAGCAGAGTAAGACTTCATTTCAAAAAATGGGAAGAACAGCAAACATTTTAACTAGCTCAGATAAGCATCCTCCTTCACAAGGAAAAGCCGCATTCAGAAAATGAATCACGTTCCCAACCTTAAGAGCCAGGTTCTGACTGCTGTAACGCAGCCCCAGAGGGGATCAGTCTGTGAGTTCCTGTCCCCTCAGTGTGGAAATGCTCCCTCCTTCAGGACCTTAATTTTCACCAGCAGGAAACTCTCACCTGGAATCAGCTTAAGGTTCTGATCTAAATAAACTGTCAAAATCATAAACCCATTCTCCAGAAGACAACCCAAACAAGTCTTTCTCTAAACCTGAACTAAACAGATTTTTTTAGAGGGAGTCTAGCTCTGTTGCCCAGGCTGGAGTGCAGTGGCGTGATCTCGGCTCACTGCAGCCCCCACCTCCCGGGTTCAAGCAGTTCTCCTGCCTC
