# Supplementary material for: RNA-seq reveals the diverse effects of substrate stiffness on epidermal ovarian cancer cells
Source: Aging (Albany NY). 2020 Oct 22;12(20):20493–511. doi: 10.18632/aging.103906 (PMC7655203; doi:10.18632/aging.103906)
Supplement: Supplementary Table 2 [file aging-12-103906-s002..docx]

| **Supplementary Table 2. Co-expression analysis between lncRNA and key targets** | | |
| --- | --- | --- |
| Co-expression with PLEC | | |
| LncRNA | Cor-Score | P-value |
| NONHSAT000101 | -0.973 | 0.001051514 |
| NONHSAT004775 | -0.854 | 0.030294033 |
| NONHSAT004796 | 0.819 | 0.046312478 |
| NONHSAT009851 | -0.863 | 0.026960936 |
| NONHSAT013041 | -0.857 | 0.029187593 |
| NONHSAT015091 | 0.969 | 0.001468974 |
| NONHSAT015217 | -0.96 | 0.002396222 |
| NONHSAT017254 | 0.907 | 0.012519683 |
| NONHSAT017255 | -0.846 | 0.033602594 |
| NONHSAT018984 | 0.934 | 0.006465389 |
| NONHSAT022470 | 0.843 | 0.035137423 |
| NONHSAT036494 | 0.883 | 0.019816532 |
| NONHSAT040643 | 0.903 | 0.013652356 |
| NONHSAT041031 | 0.878 | 0.02155811 |
| NONHSAT047181 | 0.843 | 0.035109417 |
| NONHSAT055342 | -0.895 | 0.015986455 |
| NONHSAT060290 | 0.835 | 0.038536943 |
| NONHSAT060381 | 0.874 | 0.022865594 |
| NONHSAT060382 | -0.97 | 0.001334879 |
| NONHSAT061240 | 0.849 | 0.032642227 |
| NONHSAT064303 | 0.925 | 0.008167524 |
| NONHSAT075745 | 0.907 | 0.012655744 |
| NONHSAT076385 | -0.962 | 0.002187823 |
| NONHSAT076726 | -0.857 | 0.029187593 |
| NONHSAT084830 | -0.817 | 0.046985372 |
| NONHSAT084834 | -0.857 | 0.029187577 |
| NONHSAT087761 | 0.857 | 0.029387273 |
| NONHSAT087866 | 0.874 | 0.022906448 |
| NONHSAT089478 | -0.873 | 0.02302466 |
| NONHSAT091632 | 0.844 | 0.034394833 |
| NONHSAT092802 | 0.822 | 0.044817763 |
| NONHSAT099079 | 0.894 | 0.016221143 |
| NONHSAT099905 | -0.857 | 0.029187791 |
| NONHSAT116180 | 0.891 | 0.017265503 |
| NONHSAT119300 | 0.838 | 0.03711419 |
| NONHSAT120487 | 0.848 | 0.03282487 |
| NONHSAT122235 | -0.953 | 0.003204202 |
| NONHSAT129220 | 0.892 | 0.016721216 |
| NONHSAT133696 | 0.914 | 0.010823617 |
| NONHSAT133922 | -0.842 | 0.035653061 |
| NONHSAT136925 | 0.881 | 0.020527325 |
| NONHSAT138078 | 0.879 | 0.020957069 |
| NONHSAT145933 | 0.87 | 0.02414139 |
| NONHSAT148828 | 0.921 | 0.009174294 |
| NONHSAT149677 | -0.857 | 0.029187604 |
| NONHSAT149685 | 0.919 | 0.009503527 |
| NONHSAT152962 | -0.827 | 0.042232343 |
| NONHSAT155650 | 0.96 | 0.002404881 |
| NONHSAT156769 | 0.903 | 0.013530578 |
| NONHSAT156973 | 0.903 | 0.013773064 |
| NONHSAT159447 | 0.987 | 0.000270456 |
| NONHSAT161781 | 0.835 | 0.038550266 |
| NONHSAT162929 | 0.903 | 0.013578087 |
| NONHSAT163994 | -0.886 | 0.018805741 |
| NONHSAT168171 | 0.86 | 0.028033393 |
| NONHSAT168466 | 0.874 | 0.02277596 |
| NONHSAT171662 | -0.857 | 0.029067903 |
| NONHSAT173818 | -0.986 | 0.000283588 |
| NONHSAT175268 | 0.838 | 0.037401493 |
| NONHSAT175322 | -0.857 | 0.029187593 |
| NONHSAT177304 | 0.838 | 0.037401493 |
| NONHSAT178715 | -0.831 | 0.040317464 |
| NONHSAT180470 | 0.826 | 0.043007317 |
| NONHSAT183353 | -0.857 | 0.029187593 |
| NONHSAT183824 | -0.857 | 0.029187593 |
| NONHSAT185326 | -0.857 | 0.029187586 |
| NONHSAT185865 | -0.918 | 0.009823554 |
| NONHSAT188900 | 0.96 | 0.002390832 |
| NONHSAT191567 | 0.836 | 0.038020902 |
| NONHSAT193625 | -0.85 | 0.032055233 |
| NONHSAT196371 | -0.857 | 0.029187445 |
| NONHSAT202369 | 0.816 | 0.047805262 |
| NONHSAT209970 | 0.816 | 0.047799732 |
| NONHSAT215424 | -0.881 | 0.020292214 |
| NONHSAT217250 | -0.857 | 0.029187593 |
| NONHSAT222374 | -0.834 | 0.039097927 |
| NONHSAT223418 | 0.827 | 0.042097559 |
| Co-expression with TNS2 | | |
| NONHSAT000101 | -0.825 | 0.043247239 |
| NONHSAT004796 | 0.941 | 0.005092142 |
| NONHSAT015217 | -0.958 | 0.002593785 |
| NONHSAT017254 | 0.967 | 0.001573223 |
| NONHSAT017255 | -0.976 | 0.000865135 |
| NONHSAT036494 | 0.933 | 0.006638016 |
| NONHSAT041031 | 0.878 | 0.021440997 |
| NONHSAT044052 | -0.866 | 0.025616656 |
| NONHSAT055342 | -0.983 | 0.000412458 |
| NONHSAT060290 | 0.985 | 0.000331839 |
| NONHSAT060382 | -0.936 | 0.006022788 |
| NONHSAT061079 | -0.812 | 0.049551243 |
| NONHSAT061240 | 0.998 | 0.00000668 |
| NONHSAT068736 | 0.892 | 0.016967989 |
| NONHSAT072995 | 0.909 | 0.012121859 |
| NONHSAT076385 | -0.956 | 0.002897184 |
| NONHSAT079674 | 0.84 | 0.036254629 |
| NONHSAT087866 | 0.846 | 0.033867414 |
| NONHSAT091631 | -0.891 | 0.017249002 |
| NONHSAT091632 | 0.978 | 0.000709874 |
| NONHSAT092802 | 0.974 | 0.001008251 |
| NONHSAT099079 | 0.983 | 0.000421132 |
| NONHSAT102748 | -0.863 | 0.026729222 |
| NONHSAT102871 | -0.858 | 0.029006672 |
| NONHSAT116180 | 0.906 | 0.012945104 |
| NONHSAT118445 | 0.924 | 0.008484017 |
| NONHSAT119300 | 0.894 | 0.016226172 |
| NONHSAT122235 | -0.966 | 0.001755022 |
| NONHSAT127848 | -0.964 | 0.001871002 |
| NONHSAT133696 | 0.951 | 0.003613746 |
| NONHSAT145933 | 0.886 | 0.018830723 |
| NONHSAT148514 | -0.842 | 0.03544145 |
| NONHSAT148828 | 0.861 | 0.02779623 |
| NONHSAT149685 | 0.945 | 0.004517873 |
| NONHSAT152962 | -0.941 | 0.005096588 |
| NONHSAT154958 | -0.837 | 0.037836917 |
| NONHSAT156769 | 0.935 | 0.006283589 |
| NONHSAT159447 | 0.886 | 0.018736054 |
| NONHSAT162895 | 0.844 | 0.034433292 |
| NONHSAT162929 | 0.925 | 0.008195479 |
| NONHSAT163994 | -0.855 | 0.02997633 |
| NONHSAT169584 | -0.928 | 0.007570812 |
| NONHSAT173818 | -0.814 | 0.048697882 |
| NONHSAT175268 | 0.977 | 0.000799383 |
| NONHSAT177304 | 0.977 | 0.000799383 |
| NONHSAT184582 | -0.9 | 0.014479002 |
| NONHSAT185114 | -0.84 | 0.036168856 |
| NONHSAT185865 | -0.982 | 0.000483281 |
| NONHSAT188900 | 0.928 | 0.00756654 |
| NONHSAT190893 | -0.923 | 0.008683776 |
| NONHSAT191567 | 0.996 | 0.0000202 |
| NONHSAT193625 | -0.825 | 0.043278992 |
| NONHSAT198622 | -0.96 | 0.002349132 |
| NONHSAT203904 | -0.942 | 0.00494542 |
| NONHSAT215424 | -0.963 | 0.001977939 |
| NONHSAT216190 | -0.821 | 0.04508125 |
| NONHSAT223418 | 0.93 | 0.007250485 |
