# Supplementary material for: RNA-seq reveals the diverse effects of substrate stiffness on epidermal ovarian cancer cells
Source: Aging (Albany NY). 2020 Oct 22;12(20):20493–511. doi: 10.18632/aging.103906 (PMC7655203; doi:10.18632/aging.103906)
Supplement: Supplementary Table 1 [file aging-12-103906-s003..pdf]

SUPPLEMENTARY TABLE

Supplementary Table 1. Summary of sequencing results output (N=2).

Sample Name	Reads.Num	Base.Num	Q20.Base.Num(Ratio)	Q30.Base.Num(Ratio)	GC.content	N.base.Num (Ratio)	Read.Length
H2	44440069	6666010350	6593749485(98.92%)	6459851212(96.91%)	46.47%	57069(0.00%)	150
H2	44440069	6666010350	6489819867(97.36%)	6246485767(93.71%)	47.21%	94714(0.00%)	150
C2	47842892	7176433800	7092757975(98.83%)	6941223461(96.72%)	45.81%	77453(0.00%)	150
C2	47842892	7176433800	6976098581(97.21%)	6701906563(93.39%)	46.56%	136813(0.00%)	150
L1	40890973	6133645950	6062947516(98.85%)	5935192199(96.76%)	45.07%	57346(0.00%)	150
L1	40890973	6133645950	5951631882(97.03%)	5707000804(93.04%)	45.74%	101164(0.00%)	150
C1	44666424	6699963600	6619834309(98.80%)	6475623115(96.65%)	45.40%	59622(0.00%)	150
C1	44666424	6699963600	6479745620(96.71%)	6187726666(92.35%)	46.14%	106154(0.00%)	150
L2	39983412	5997511800	5926191632(98.81%)	5796582141(96.65%)	45.39%	54755(0.00%)	150
L2	39983412	5997511800	5823812613(97.10%)	5589071350(93.19%)	46.12%	90218(0.00%)	150
H1	46955773	7043365950	6959569622(98.81%)	6807760164(96.65%)	45.97%	61925(0.00%)	150
H1	46955773	7043365950	6836222218(97.06%)	6558983836(93.12%)	46.71%	107234(0.00%)	150
