# Supplementary material for: RNA-seq reveals the diverse effects of substrate stiffness on epidermal ovarian cancer cells
Source: Aging (Albany NY). 2020 Oct 22;12(20):20493–511. doi: 10.18632/aging.103906 (PMC7655203; doi:10.18632/aging.103906)
Supplement: Supplementary Figures [file aging-12-103906-s004..pdf]

SUPPLEMENTARY FIGURES

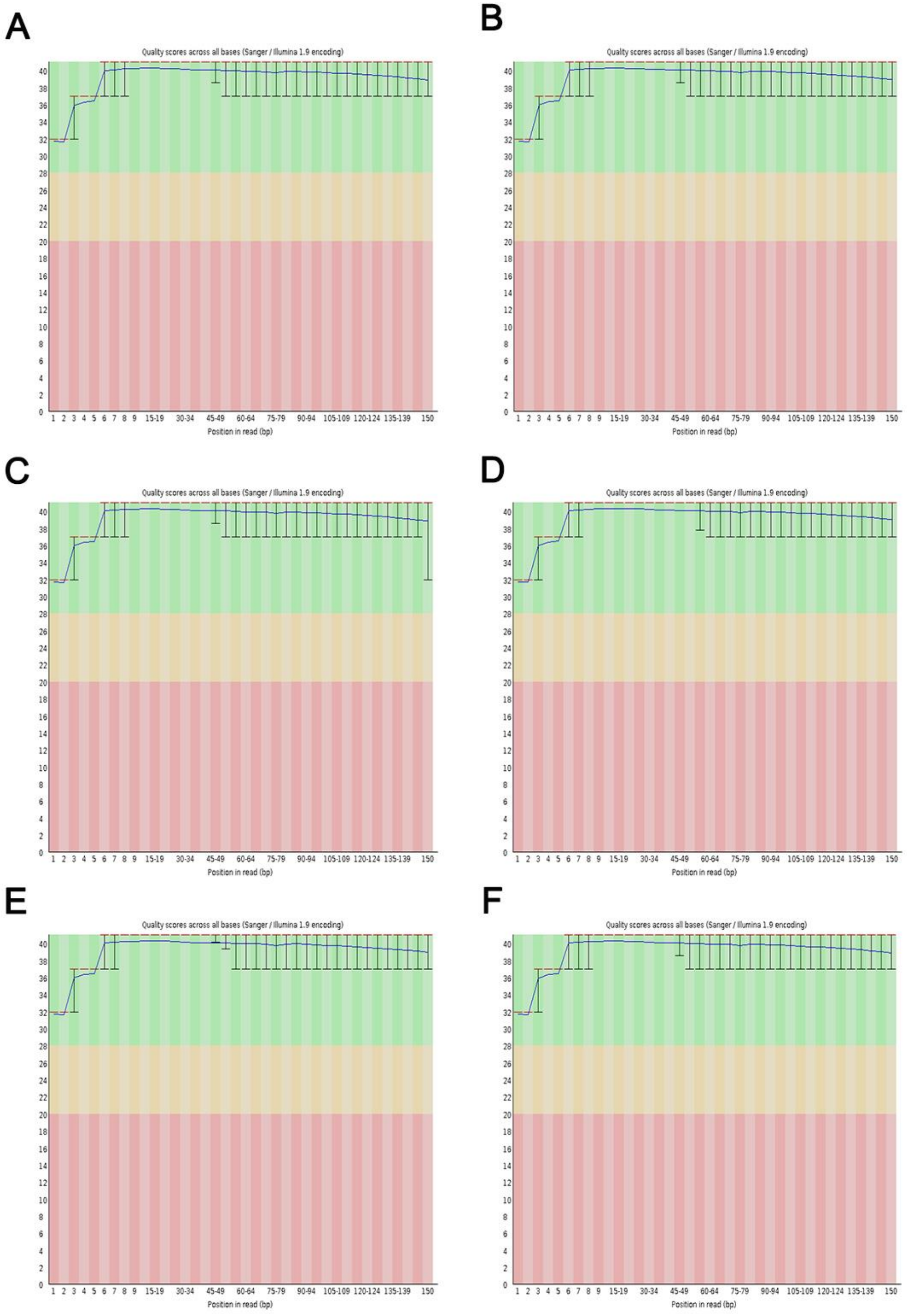

Supplementary Figure 1. The BoxWhisker map shows all the base mass ranges in the FastQ sequencing file. The Y axis shows the base mass score, and the higher the score is, the better the base sequencing quality is. The background color of the map divides the Y axis into regions with high sequencing base quality (green), qualified regions (orange) and poor-quality regions (red). (**A**–**F**) represented control group (6 kPa) 1, 2, high group (60 kPa) 1, 2 and low group (1 kPa) 1, 2, respectively.

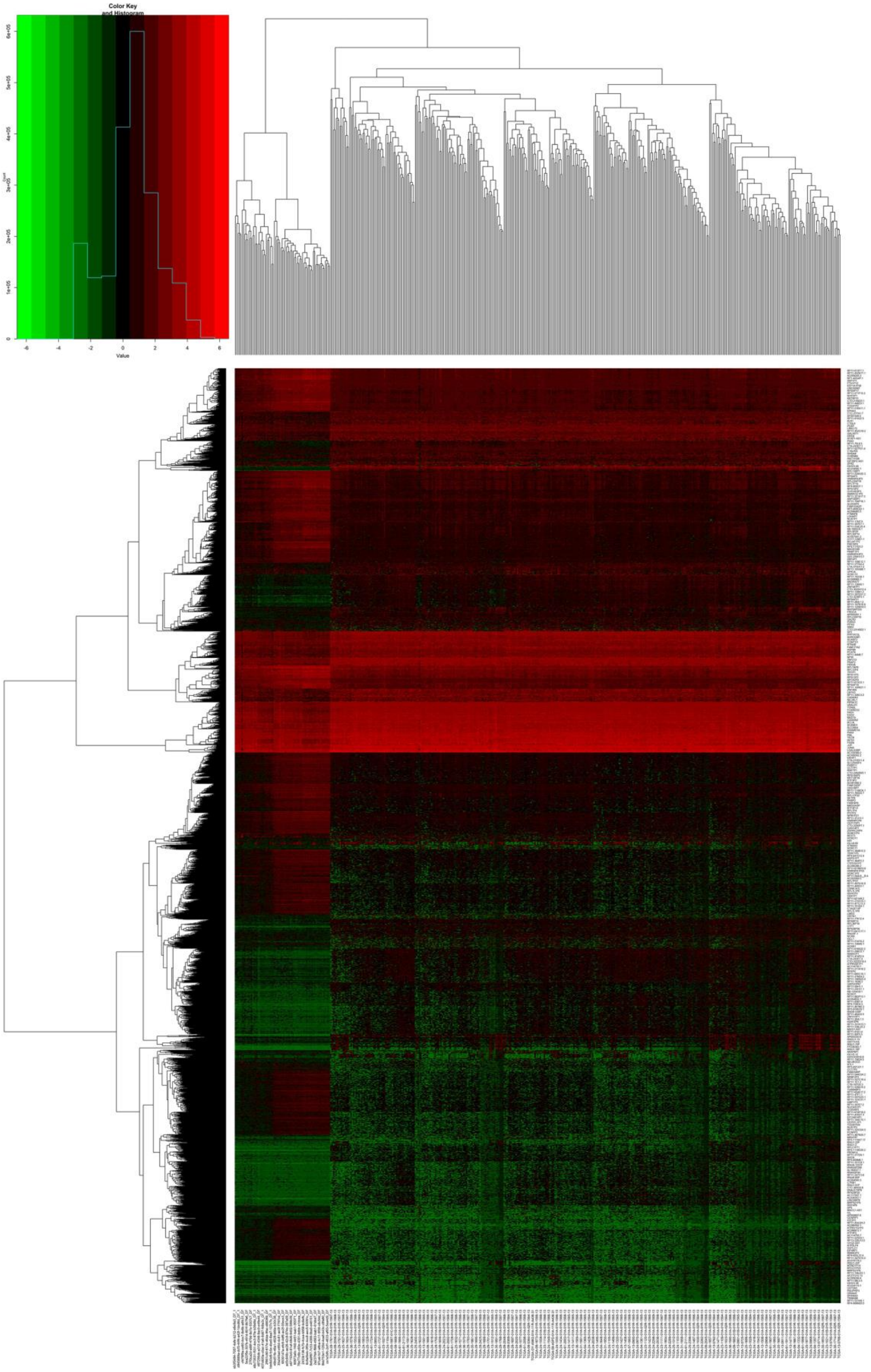

Supplementary Figure 2. Heatmap of TCGA EOC data. Red corresponds to up-regulation and green to down-regulation.
